# Comment on “Establishing a Porcine Model of Small for Size Syndrome following Liver Resection”

**DOI:** 10.1155/2018/4915817

**Published:** 2018-05-22

**Authors:** Antonios Athanasiou, Eleftherios Spartalis, Mairead Hennessy, Michael Spartalis, Emmanouil Pikoulis

**Affiliations:** ^1^Department of Upper Gastrointestinal Surgery, University Hospitals Birmingham NHS Foundation Trust, Birmingham, UK; ^2^Laboratory of Experimental Surgery and Surgical Research, University of Athens, Medical School, Ag. Thoma 15B, 11527 Athens, Greece; ^3^Department of Surgery, Mercy University Hospital, Grenville Place, Cork, Ireland; ^4^First Surgery Department, Laikon General Hospital, National and Kapodistrian University of Athens, Athens, Greece

We read with great interest the article by Golriz et al. [1] published in the August 2017 issue of Canadian Journal of Gastroenterology and Hepatology. The aim of this research study was to establish a porcine model of small for size syndrome (SFSS). The authors divided 24 Landrace pigs into 3 groups according to the remnant liver volume; group A, group B, and group C underwent liver resection with a remnant liver volume of 50%, 25%, and 15%, respectively. Gorliz and his colleagues conclude that 75% liver resection in porcine model results in SFSS. This is a very interesting research manuscript in our understanding regarding the establishment of a porcine model of small for size syndrome after extended hepatectomy. However, there are some questions which demand further consideration.

To start with, this study does not include either any information as regards the monitoring of portal vein flow and pressure or any measurement of hepatic artery flow and pressure. Animal experiments managed to prove that portal hemodynamic changes are considered to be the most important mechanisms of posthepatectomy liver failure [2–6]. Same results have been proven in clinical practice [7–9]. For this reason we believe that is not only insufficient, but also unreliable to assess precisely the 3 surgical models that the authors studied. Furthermore, recent publications demonstrated that reliable porcine models for SFSS have to include apart from the hemodynamic measurements of portal vein and hepatic artery the hepatic venous pressure gradient (HVPG) [10, 11]. Significant increase in HVPG immediately after liver resection and 7 days after hepatectomy has strong correlation with the manifestation of SFSS [10, 11].

Furthermore, our literature review and our recent experiments for the investigation of SFSS in porcine model showed that only after 80% liver resection, this model proved to be appropriate for the study of SFSS [5, 12–18]. The remnant liver volume after this resection in combination with portal hyperperfusion and hypertension results in a significant reduction of the hepatic portal vascular bed, which means dramatic increase of pressure and flow per grammar of liver tissue [19]. This condition leads to hepatic sinusoidal injury and severe hepatocellular damage. The histopathological and laboratory findings, survival rate, liver regeneration, and apoptosis and also the portal hemodynamic changes 7 days after 80% liver resection are similar to the clinical manifestations of SFSS [12–16].

Last but not least, Golriz et al. report at the conclusion of the manuscript that 75% liver resection in porcine model results in SFSS, while 85% liver resection causes irreversible liver failure. However, this study has not proved that 85% liver resection in porcine model creates nonreversible liver failure. This is because the researches did not apply any measures for the improvement of liver function postoperatively, which means that this is only a hypothesis and not a conclusion [20]. As many studies proved during the last decade, porcine model after 85% liver resection could survive for more than 14 days postoperatively [5, 21, 22].

According to the recent studies, it was demonstrated that hypoxia probably plays a major role for the triggering of liver regeneration [23]. Greater rapid hypertrophy after liver resection could be explained not only by portal hypertension and hyperflow, but also by hypoxia which reverse arterial buffer response [13, 24, 25]. The histological evaluation of hepatocyte proliferation in the 3 groups by measuring Ki-67 proliferative index and nuclear factor kappa-beta expression should be really interesting. By doing so, Golriz et al. should be able to evaluate more accurately the liver regeneration among the 3 groups [1].
